# Ten-year atherosclerosis cardiovascular disease (ASCVD) risk score and its components among an Iranian population: a cohort-based cross-sectional study

**DOI:** 10.1186/s12872-022-02601-0

**Published:** 2022-04-09

**Authors:** Fatemeh Zibaeenejad, Seyyed Saeed Mohammadi, Mehrab Sayadi, Fatemeh Safari, Mohammad Javad Zibaeenezhad

**Affiliations:** 1 Cardiovascular Research Center, Shiraz University of Medical Sciences, Khalili St., Shiraz, Iran; 2 Department of Physiology, Shahid Sadoughi University of Medical Sciences, Yazd, Iran

**Keywords:** Atherosclerotic cardiovascular disease, ASCVD, Gender, Age, Risk score

## Abstract

**Background:**

Atherosclerotic cardiovascular disease (ASCVD) continues to be the first cause of mortality globally. Effective preventive strategies require focused efforts to clarify ASCVD risk factors in different subgroups of a population. This study aimed to identify individuals at higher risk of ASCVD among Shiraz University employees to guide decision-making for primary prevention.

**Methods:**

This cohort-based cross-sectional study was conducted on data of 1191 participants (25–70 years old) from Shiraz University employees selected by systematic random sampling. The 10-year ASCVD risk was calculated with an ASCVD risk score estimator developed by the American College of Cardiology/American Heart Association (ACC/AHA). To analyze the data, descriptive and chi-square tests were used. All statistical analyses were conducted using the SPSS version 16.0 software. The *p*-value < 0.05 was considered a significant level.

**Results:**

This study demonstrated that 75.3% of the participants had low risk scores, whereas 13.2% and 2.5% of them had intermediate and high risk scores, respectively. Additionally, it revealed that among women 93.7%, 2.7%, and 0.6% had low intermediate and had high risk scores, respectively, whereas among men, 61.5%, 21.1%, and 3.9% had low intermediate and high risk scores, respectively. Based on the results of the chi-square test, men were significantly more prone to ASCVD (38.5%) than women (6.3%) were. Interestingly, 40.9% of known cases of hypertension had uncontrolled blood pressure, and 62.5% of individuals without any history of hypertension, who were considered new cases of hypertension, had abnormal blood pressure. Furthermore, 38.5% of diabetic patients and 1.6% of people who did not have a history of diabetes had abnormal serum fasting blood sugar**.**

**Conclusion:**

It was revealed that nearly 15.7% of participants were at intermediate and high risk of developing ASCVD in the next 10 years with greater risk in men. Considerably, some of hypertensive and diabetic participants had uncontrolled blood pressure and blood sugar levels, respectively. New cases of diabetes and hypertension were also recognized in our study. Therefore, to address the primary prevention of ASCVD in this population, it is necessary to have plans for targeted interventions, which can be effective in modifying their risk factors.

## Introduction

Atherosclerosis is a slowly progressive disease characterized by hardening and narrowing of arteries due to accumulation of lipids and remodeling of extracellular matrix. It is an inflammatory process, and both innate and adaptive immune systems play a role in all of its stages, including formation and progression of fibro fatty lesion and final complications [1–3].

Atherosclerosis is the prime root of cardiovascular events contributing to the most considerable number of morbidity and mortality worldwide and will remain the major cause of death by 2030 [4, 5]. Clinical presentation of ASCVD ranges from acute coronary syndrome to a history of myocardial infarction, stable or unstable angina, coronary or other arterial revascularization, stroke, transient ischemic attack, or peripheral arterial disease, which is assumed to be of an atherosclerotic origin [6].

The incidence of cardiovascular disease (CVD) has rapidly increased in developing countries. Successful prevention of CVD is desperately needed to decrease this substantial burden and relevant economic costs [7, 8]. Despite remarkable advancements in the treatment of CVD, it is definitely important to clarify the root causes of CVD to develop effective preventive strategies [9–11].

Although some cardiovascular risk factors, such as age, sex, familial history, and ethnicity, are not manageable to direct therapeutic intervention, it is widely believed that they are important for stratifying risk factors of CVD [12]. In addition, emerging evidence suggests that modifiable risk factors are central to the pathogenesis of CVD. For example, there is adequate evidence that systolic and diastolic blood pressures (BP) are firmly related to CVD [13]. Being diagnosed with diabetes mellitus (DM) is related to a considerably higher cardiovascular risk [14, 15].

Abnormal cholesterol and triglyceride levels are the cause for approximately half of a population’s risk of developing ASCVD [16, 17]. The extent of multiplicative interactions of risk factors for developing CVD is so important that the need for developing algorithms predicting the ASCVD is absolutely essential [18, 19].

In 1976, the first coronary heart disease (CHD) risk equations were released by Framingham Heart Study investigators [20]. Since then, various algorithms for CVD risk calculation have been published to be used by public health and in clinical practice. In the United States, the Framingham general CVD equations and Pooled Cohort Equations (PCE) for ASCVD, the QRISK in the United Kingdom, and the Systematic Coronary Risk Evaluation model in Europe are among the important examples to mention [21]. Estimation of an individual’s 10-year ASCVD risk helps clinicians to clarify the intensity of preventive strategies, to maximize desired outcome and minimize eventual harm due to overtreatment [22, 23].

There are regional and national differences in the incidence and mortality of ASCVD, which are due to the differences in the prevalence of ASCVD risk factors and access to high-quality health services. However, the role of genetic factors associated with different races should be considered. In Iran, the prevalence of cardiovascular diseases risk factors has increased during the past few decades owing to socioeconomic and cultural changes, such as, unhealthy diets combined with insufficient physical activity, and shortage of access to health care. In Iran, ischemic heart disease and stroke, which are mainly due to atherosclerosis, have been regarded the leading causes of death and disability [24]. Hence, successful prevention of ASCVD is obviously needed to reduce the current burden and economic cost of these diseases. There are few epidemiological data on the prevalence of ASCVD risk factors among the Iranian subpopulation. Therefore, this study aimed to estimate the prevalence of ASCVD risk factors among Shiraz University employees using a 10-year ASCVD risk predicting estimator.

## Materials and methods

### Study population

This study enrolled 1191 Shiraz university employees and their spouses between the ages of 25 and 70 who were enlisted in a prospective cohort study in Shiraz (the capital of Fars province in southwest Iran) that began in 2018 and inducted 7643 participants (Shiraz Cohort Heart Study, SCHS) [25, 26]. Additionally, 773 of 1191 participants were between 40 and 70 years old. The sample size was determined using the formulas $n = \frac{{z^{2} p(1 - p)}}{{d^{2} }}\;{\text{and}}\;n^{\prime } = \frac{n}{{1 + \frac{n}{N}}}$ with the following assumptions: *p* = 0.1, d = 0.015, 95% CI, and an infinite size of staff.

Participants who had a cerebrovascular accident, transient ischemic attack, coronary artery disease, carotid artery disease, peripheral vascular disease, a positive exercise tolerance test, a typical angina history, congestive heart failure, or electrocardiography with evidence of myocardial infarction or ischemic heart disease were excluded. The study was approved by the Shiraz University of Medical Sciences' research ethics committee (IR.SUMS.MED.REC.1400.141).

### Risk factor definitions and 10-year risk stratification

Participants were classified as normal or abnormal based on the presence or absence of risk factors such as a body mass index (BMI) > 25, high total cholesterol, low HDL, high LDL, high TG, diastolic, and systolic blood pressure, and a history of diabetes or smoking. The 10-year ASCVD risk was estimated using the ASCVD risk score estimator as per the 2019 ACC/AHA guideline. This guideline presents an evidence-based approach to comprehensive risk factor management, reducing CVD incidence [27].

Gender, age, race, cholesterol profile, blood pressure, use of antihypertensive therapy, family history of DM, and smoking history were required to calculate the 10-year risk score for ASCVD. The results were classified by risk category (low, borderline, intermediate, and high risk) and gender. Individuals were informed of the findings and encouraged to take preventative measures to lower risks.

The following definitions of abnormal levels were used in this study:

Hypertension (HTN): if diagnosed clinically, or if they had a systolic blood pressure of ≥ 130 mmHg either-or a diastolic blood pressure of ≥ 80 mmHg, or if they used antihypertensive medications.

Triglyceride (TG) levels of ≥ 200 mg/dl, total cholesterol levels of ≥ 200 mg/dl, HDL cholesterol levels of ≥ 40 mg/dl for men and ≥ 50 mg/dl for women, and LDL cholesterol levels of ≥ 160 mg/dl were considered abnormal.

The history of smoking included cigarettes, cigars, and water pipes.

DM if it was previously diagnosed in their clinical history.

A BMI of > 25 was considered abnormal.

Additionally, patients were classified as low, borderline, intermediate, or high-risk based on their risk scores:

- Low-risk: < 5%
- Borderline-risk: 5–7.5%
- Intermediate-risk: ≥ 7.5– < 20%
- High-risk: ≥ 20%

### Statistical analysis

This study employed descriptive statistics and chi-square tests (to determine statistically significant differences). The data were analyzed using the statistical package for social sciences (SPSS) software for Windows (v. 16, Chicago, SPSS Inc.). A *p*-value of < 0.5 was deemed significant.

## Results

### Demographic features

Table 1 summarizes the demographic characteristics of 1191 Shiraz University employees and their spouses, ranging from 25 to 70 years old (mean: 44.28 ± 8.94).

**Table 1:** Demographic characteristics of 1191 Shiraz University employees who participated in this study (25–70 years old)

Variable	Total (n = 1191)	Male (n = 638)	Female (n = 553)
Age group			
25–30	37 (3.1)	14 (2.2)	23 (4.2)
30–35	153 (12.8)	70 (11.0)	83 (15.0)
35–40	285 (23.9)	140 (21.9)	145 (26.2)
40–45	232 (19.5)	119 (18.7)	113 (20.4)
45–50	188 (15.8)	111 (17.4)	77 (13.9)
50–55	147 (12.3)	85 (13.3)	62 (11.2)
55–60	83 (7.0)	55 (8.6)	28 (5.1)
60–65	46 (4.9)	26 (4.1)	20 (3.6)
Above 65	20 (1.7)	18 (2.8)	2 (0.4)

Data are presented as number and percentage of participants

### Cardiovascular risk factors' prevalence

Serum lipid analysis revealed that 37.03% of Shiraz University employees had abnormal cholesterol levels (≥ 200 mg/dl), while 47.1% had low HDL levels (< 40 mg/dl for men and < 50 mg/dl for women). Additionally, 7.69% of individuals had elevated LDL (≥ 160 mg/dl) and 17.6% had elevated TG (≥ 200 mg/dl) serum levels.

In terms of serum FBS and diabetes mellitus status, 5.1% of participants had elevated FBS (> 126 mg/dl). Moreover, 9.2% had DM based on their family history.

In our study, 19.4% and 39.6% of participants exhibited abnormal systolic and diastolic blood pressures, respectively.

According to the data, 65.6% had an elevated BMI (≥ 25). Additionally, 11.5% of participants were smokers.

**Fig. 1:**
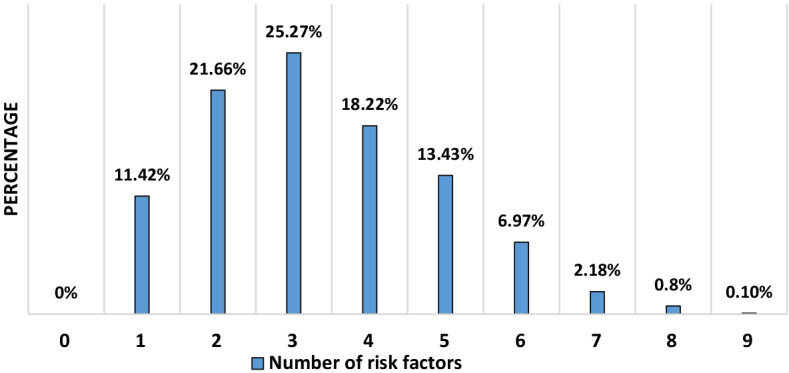
The prevalence of a variety of risk factors (abnormal total cholesterol, HDL, LDL, TG, systolic and diastolic BP, FBS, BMI, as well as a history of smoking) among Shiraz University employees (25–70 years) (n = 1191)

The prevalence of the aforementioned cardiovascular risk factors is shown in Table 2 and Fig. 1. Furthermore, it is worth noting that all participants had at least one of the cardiovascular disease risk factors listed in Table 2.

**Table 2:** The prevalence of risk factors for cardiovascular disease in Shiraz University employees (n = 1191) (25–70 years)

Variables	Count	Percentage
Total cholesterol		
Normal	750	63
Abnormal (≥ 200)	441	37
HDL		
Normal	630	52.9
Abnormal (< 40 in men and < 50 in female)	561	47.1
LDL		
Normal	1101	92.31
Abnormal (≥ 160)	90	7.69
TG		
Normal	981	82.4
Abnormal (≥ 200)	210	17.6
FBS		
Normal	1130	94.9
Abnormal (≥ 126)	61	5.1
Systolic BP		
Normal	960	80.6
Abnormal (≥ 130 mmHg)	231	19.4
Diastolic BP		
Normal	719	60.4
Abnormal (≥ 80 mmHg)	472	39.6
BMI		
Normal (18.5 ≤ BMI < 25)	410	34.4
Overweight (25 ≤ BMI < 30)	538	45.2
Obese (≥ 30)	243	20.4
Smoking	137	11.5
Antihypertensive treatment	109	9.2
DM		
Normal	1082	90.8
Based on history	109	9.2

HDL, high-density lipoprotein; LDL, low-density lipoprotein; TG, triglyceride; FBS, fasting blood sugar; BMI, body mass index; BP, blood pressure; DM, diabetes mellitus

### ASCVD risk score

Considering the five risk factors involved in ASCVD risk score estimator (history of DM and smoking, as well as abnormal ranges for total cholesterol, HDL, and systolic blood pressure), 18.8% (n = 224) of participants were risk-free, 47.3% (n = 563) had one risk factor, 26% (n = 310) had two risk factors, 6.7% (n = 80),1.2% (n = 14) and 0.1% (n = 1) exhibited three, four, and five risk factors, respectively.

HTN and DM are two modifiable independent risk factors for ASCVD. As a result, the prevalence of these risk factors was determined among our study's participants. A total of 127 participants were known cases of HTN, and 40.9% (52 participants) had abnormal BP (uncontrolled BP), which should be closely monitored to ascertain the probable cause or causes. Additionally, 59.1% (75 individuals) of participants had normal BP (controlled bp) and were required to maintain their current antihypertensive therapy and routine follow-up. Among those without a history of HTN (1064 individuals), 42.6 percent (453 individuals) had abnormal BP and were considered new HTN cases.

In terms of diabetes prevalence, our data show that 109 participants were known cases of DM, with 61.5% (67 individuals) exhibiting normal FBS (controlled diabetes). However, 38.5% (42 individuals) had abnormal FBS (uncontrolled diabetes), which should be closely monitored. Furthermore, 1.6% of the individuals with no history of DM demonstrated abnormal FBS, so they were advised to undergo additional tests for DM diagnosis (Table 3).

**Table 3:** Prevalence of known and unknown hypertension (HTN) and diabetes mellitus (DM)

Cases with diagnosed HTN	Yes (n = 127)	Abnormal BP	Normal BP
		52 (40.9)	75 (59.1)
	No (n = 1064)	453 (42.6)	611 (57.4)
Cases with diagnosed DM	Yes (n = 109)	Abnormal FBS	Normal FBS
		42 (38.5)	67 (61.5)
	No (n = 1062)	17 (1.6)	1045 (98.4)

Data are presented as number and percentage of participants

BP, blood pressure; FBS, fasting blood sugar

The ASCVD risk scores of 1191 participants (25–70 years old) in this study ranged from 0.05 to 48.76% (mean: 4.88 ± 3.12%). Among the participants, 81.2% (n = 967) were classified as low-risk, 8.3% (n = 99) borderline-risk, 8.7% (n = 104) intermediate-risk, and 1.8% (n = 21) high-risk. Furthermore, among 773 participants (40–70 years old), risk scores ranged from 0.14 to 47.13% (mean: 4.28 ± 5.31%). A total of 75.3% (n = 582) of all participants were classified as low-risk, 9.1% (n = 70) borderline-risk, 13.2% (n = 102) intermediate-risk, and 2.5% (n = 19) high-risk (Table 4).

**Table 4:** Atherosclerotic cardiovascular disease (ASCVD) risk score prevalence in two Shiraz University employees age groups

Age	Risk score	Count	Gender		*P* value
			Male	Female	
40–70 year	Low (< 5%)	582 (75.3)	271 (61.5)	311 (93.7)	< 0.001
	Borderline (5–7.5%)	70 (9.1)	60 (13.6)	10 (3)	
	Intermediate (7.5–20%)	102 (13.2)	93 (21.1)	9 (2.7)	
	High (≥ 20)	19 (2.5)	17 (3.9)	2 (0.6)	
25–70 year	Low (< 5%)	967 (81.2)	442 (69.3)	525 (94.9)	< 0.001
	Borderline (5–7.5%)	99 (8.3)	82 (12.9)	17 (3.1)	
	Intermediate (7.5–20%)	104 (8.7)	95 (14.9)	9 (1.6)	
	High (≥ 20)	21 (1.8)	19 (3.0)	2 (0.4)	

n = 1191 (25–70 year olds) and n = 773 (40–70 year olds). Data are presented as number and percentage of participants

According to the data analysis of the participants aged 40–70, 93.7% of females were classified as low-risk, 2.7% intermediate-risk, and 0.6% high-risk. At the same time, 61.5% of males were classified as low-risk, 21.1% intermediate-risk, and 3.9% high-risk. According to the chi-square test, males are significantly more likely than females to develop ASCVD (P < 0.001) (Table 4).

In the 25–70 age group, 94.9% of females were classified as low-risk, 1.6% intermediate-risk, and 0.4% high-risk. Among the male participants, 69.3% were low-risk, 14.9% intermediate-risk scores, and 3% high-risk. According to the chi-square test results, males are significantly more likely than females to develop ASCVD (P < 0.001) (Table 4).

By comparing the ASCVD risk score of 773 Shiraz university staff (40–70 years old, mean: 49.08 ± 7.00) to that of 6452 Shiraz cohort study participants (40–70 years old, mean: 52.51 ± 8.04), we concluded that 62.5% of Shiraz cohort study participants were classified as low-risk, compared to 75.3% of Shiraz University employees. Additionally, 22.1% of the Shiraz cohort study population demonstrated intermediate-risk scores, while 3.4% showed high-risk scores. However, 13.2% and 2.5% of participants in our study, respectively, exhibited intermediate or high-risk scores (Table 5), indicating that Shiraz University employees are potentially less likely to develop ASCVD (*P* < 0.001) [25, 26].

**Table 5:** Comparison of ASCVD risk scores between Shiraz University employees (n = 773, aged 40–70 years old) and the Shiraz Cohort Heart Study (n = 6452, 40–70 year-olds)

	Shiraz cohort heart study (n = 6452)	Shiraz university employees (n = 773)	*P* value
Low (< 5%)	4033 (62.5)	582 (75.3)	< 0.001
Borderline (5–7.5%)	774 (12.0)	70 (9.1)	
Intermediate (7.5–20%)	1426 (22.1)	102 (13.2)	
High (≥ 20)	219 (3.4)	19 (2.5)	

Data are presented as number and percentage of participants (25, 26)

## Discussion

The aim of this study was to assess the 10-year ASCVD risk score in Shiraz University staff and to provide a guide to reduce cardiovascular risk scores in individuals with high and intermediate risk scores.

Calculation of the 10-year ASCVD risk score could be the first step in employing preventive strategies, such as lipid profile and blood pressure control [22, 23, 28]. To educate patients about means to reduce their risk other than pharmacotherapy is crucial [22, 23, 29]. In terms of accuracy, every risk estimation tool has its constraints. Thus, the use of a population-based tool to report individuals’ risk requires the consideration of their specific conditions. Moreover, it is proven that the PCE could either overestimate or underestimate the ASCVD risk for certain subgroups [30, 31]. Accordingly, healthcare providers must employ additional risk-enhancing factors to be able to effectively provide borderline- or intermediate-risk adults with reasonable guides [32]. Additionally, there are patients with borderline or intermediate 10-year risk, who are reluctant to start therapy unless being persuaded that they have increased the ASCVD risk [33].

Our study indicated that high BMI was the most common risk factor among all of the studied risk factors including HTN, DM, low HDL, high LDL, high TG, and high total cholesterol. The prevalence of obesity in our population has increased due to urbanization and industrialization as factors promoting a sedentary lifestyle as well as shifting from Mediterranean diets to western diets [34]. Thus, higher cardiovascular events will be forecasted in the future in agreement with ACC/AHA estimations. Taking measures to reduce the prevalence of this dominant risk factor in order to prevent CVD is extremely important. Furthermore, since all participants had at least one of the investigated risk factors and according to the overall high prevalence of other risk factors, management and treatment of modifiable risk factors are a considerable step.

In Iran, the prevalence of diabetes mellitus increased by 35% from 2005 to 2011, and it is estimated that in 2030, at least 9 million Iranian will be diagnosed with diabetes mellitus [35]. Our results also suggest that according to the incidence rate of high FBS (1.6%) and HTN (4.1%), screening of these risk factors must be strongly considered in primary prevention clinics. Numerous treatments are available that can reduce diabetes-induced cardiovascular complications provided that the diagnosis is made in a timely manner [36].

Epidemiological studies have demonstrated that the prevalence of HTN in Iran is 25% in women and 24% in men, which increased to 42% in the elderly [37]. There is sufficient evidence that HTN is closely associated with further cardiovascular complications [13]. Therefore, screening for early diagnosis of this silent killer is a crucial element in designing a more effective program to control this modifiable risk factor [37].

There is a strong relationship between prevalence of CVD risk factors and lifestyle. Smoking, for example, demonstrates a dose effect and undesirable interaction with other risk factors (e.g. lipids, diabetes) [38]. Smoking and smokeless tobacco (e.g. chewing tobacco) increase the risk of developing ASCVD and mortality. Secondhand smoke is a cause of ASCVD and stroke, and almost one third of deaths related to coronary heart disease are linked to smoking and being exposed to secondhand smoke. Even low level of smoking is associated with increased risks of acute myocardial infarction; therefore, reducing the number of cigarettes per day does not fully eliminate the risk. Chronic use of cigar or tobacco is linked with a persistent increase in oxidative stress and sympathetic stimulation in healthy young individuals [39].

There are few studies concerning the prevalence of ASCVD in the Iranian population. For example, Alaie Faradonbeh et al. conducted a cross-sectional study in Isfahan Province, Iran, with a population of 418 patients with DM (30–74 years old). Risk assessment was performed for all of the patients using ASCVD risk calculators. This study revealed that 28.90% of participants had a risk score < 5% and 59% had risk scores ≥ 7.5%. Furthermore, our study as well as Shiraz Cohort Heart Study, have shown that the male participants significantly had a higher ASCVD score than the female. ASCVD risk scores varied from 0.50 to 54.30%, and the mean 10-year risk of ASCVD risk score was 12.39%, which increased dramatically with age [40]. The results indicated that in the coming years, the Iranian population is going to be at higher risk of cardiovascular diseases. Motamed et al. assessed the ASCVD risk score in an Iranian population in Amol (a city in the north of Iran), with 3201 participants (40–79 years old). Based on the risk assessment by the ACC/AHA approach, we concluded that 53.5% of male participants had a 10-year risk of CVD events ≥ 10% and 28.1% of them had a 10-year risk of CVD events ≥ 20%. Moreover, 20.1% and 6.8% of women had a 10-year risk ≥ 10% and ≥ 20%, respectively [41]. In agreement with the results of the study conducted by Motamed et al., our findings revealed that male participants exhibited more tendency to develop ASCVD than female participants did.

Kandula et al. conducted a community‐based cohort study (MASALA study). It included 893 participants (40–70 years old), who were from South Asian ethnicity (at least 3 grandparents born in any South Asian country). They showed that the most common ASCVD risk factors in men and women were hyperlipidemia and HTN. The prevalence of diabetes was 28% in men and 22% in women. In addition, they found that 49% of South Asian men and 13% of women had a high 10-year risk (≥ 7.5%) [42]. In one cross-sectional study conducted by Khursheed Hassan et al., with 9885 participants (40–79 years old), the results indicated that 69.3% of all participants had an ASCVD risk score < 7.5%. Furthermore, 20.9% and 9.8% had ASCVD score ≥ 7.5 and ≥ 20%, respectively [43]. Ren et al. also conducted a retrospective study on 218 type 2 diabetic patients with diabetic kidney disease (DKD), and without known cardiovascular diseases. The 10-year ASCVD risk score was estimated using PCE, and a negative correlation has been demonstrated between glomerular filtration rate and the 10-year ASCVD risk score in patients with DKD. In their study, the median 10-year ASCVD risk score was 14.1%, and the median of the ASCVD risk score in CKD stages 1, 2, 3, and 4 was 10.9%, 12.3%, 16.5%, and 14.8%, respectively [44]. Mosepele et al. conducted a cross-sectional study on HIV-infected patients (30–50 years old) to identify the patients at higher risk of CVD. Among the patients, the mean 10-year ASCVD risk was 4%. Of all participants, 85.9% had a < 7.5% risk score and 14.1% had a ≥ 7.5% risk score [45].

More importantly, our study was able to identify the diabetic and hypertensive patients with uncontrolled blood sugar and blood pressure, which should be closely monitored to pinpoint the probable causes. Furthermore, new cases of diabetes and hypertension were also diagnosed in our study. Hence, it is mandatory to have plans for primary prevention of ASCVD in this population.

Finally, our data also show that in Shiraz University employees, intermediate and high 10-year risk scores were lower than SCHS scores. One possible explanation for the difference between the results of these two populations can be the educational and socioeconomic status; however, further data will be needed to confirm this hypothesis.

## Conclusion

From this study, we concluded that nearly three quarters of participants had a low 10-year risk score; however, 15.7% are at intermediate and high risk of developing cardiovascular diseases in the following 10 years. Thus, taking proper measures and sending preventive or therapeutic guidelines based on the 2019 ACC/AHA guideline on the primary prevention of cardiovascular diseases are vital. In addition, this study demonstrated that males had a greater tendency to develop ASCVD than females in the next 10 years, which was more than expected; therefore, it is recommended that this issue should be considered in future preventive strategies.

Furthermore, new cases were diagnosed with DM and HTN. Moreover, uncontrolled blood sugar and blood pressure in known cases of hypertension and diabetes were considered. Hence, to effectively prevent ASCVD in this population, targeted interventions are necessary to modify their risk factors.

## Data Availability

The datasets generated during and/or analyzed during the current study are not publicly available due to confidential issues but are available from the corresponding author on reasonable request.

## Abbreviations

ASCVD: Atherosclerotic cardiovascular disease
AHA: American Heart Association
ACC: American College of Cardiology
CVD: Cardiovascular disease
CHD: Coronary heart disease
BP: Blood pressure
FBS: Fasting blood sugar
HTN: Hypertension
BMI: Body mass index
DM: Diabetes mellitus
SCHS: Shiraz Cohort Heart Study
